# Dr. Gillette

**Published:** 1860-01

**Authors:** 


DR. GILLETTE, a highly respectable practitioner, physician to the Hospital for Sick Children, and to the Louis le Grand College at Paris, lately died of diphtheria, contracted, as was supposed, while in attendance upon a child suffering under that disease.

